# Subcortical shape biomarkers reveal limbic and basal ganglia damage in anti-LGI1 encephalitis

**DOI:** 10.3389/fimmu.2025.1623577

**Published:** 2025-07-04

**Authors:** Jianping Qiao, Zhishun Wang, Jiaxiang Xin, Shengjun Wang, Anning Li

**Affiliations:** ^1^ School of Physics and Electronics, Shandong Normal University, Jinan, China; ^2^ Department of Psychiatry, Columbia University, New York, NY, United States; ^3^ Magnetic Resonance (MR) Research Collaboration, Siemens Healthineers Ltd, Shanghai, China; ^4^ Department of Neurology, Qilu Hospital, Cheeloo College of Medicine, Shandong University, Jinan, China; ^5^ Department of Radiology, Qilu Hospital, Cheeloo College of Medicine, Shandong University, Jinan, China

**Keywords:** anti-LGI1 encephalitis, quantitative analysis, shape analysis, subcortical structures, shape biomarkers

## Abstract

**Introduction:**

Anti-LGI1 encephalitis is associated with disruptions in large-scale brain network functionality. Although hippocampal atrophy has been structurally characterized, the morphometric patterns of subcortical structures and their surface deformations remain poorly understood. We therefore investigated the shape abnormalities of subcortical structures and their morphological correlations in patients with anti-LGI1 encephalitis.

**Methods:**

This study included 31 patients diagnosed with anti-LGI1 encephalitis and 31 group-matched healthy controls. The mesh-based shape method was performed on the fifteen segmented subcortical structures for vertex-wise analyses. Permutation method based on general linear model was applied for statistical group comparison. Associations with disease severity and cognitive impairment were assessed in the patients. The volumetric representations of these subcortical structures were also estimated. Correlations between subcortical shape alterations and disease severity were explored.

**Results:**

Significant inward shape deformations were observed in the limbic system and basal ganglia in patients with anti-LGI1 encephalitis compared to healthy controls. Moreover, correlation analyses revealed that greater inward shape indices in the hippocampus and thalamus were associated with increased disease severity and poorer cognitive functioning, underscoring the pathological significance of these morphological alterations.

**Discussion:**

These findings indicate that precisely localized subcortical shape deformations are associated with disease severity and cognitive impairment, suggesting widespread damage of limbic system and basal ganglia in anti-LGI1 encephalitis.

## 1 Introduction

Anti–leucine-rich glioma-inactivated 1 (anti-LGI1) encephalitis is an autoimmune disorder characterized by antibodies targeting the LGI1 protein. LGI1 plays a pivotal role in maintaining brain health by regulating neuronal excitability, synaptic transmission, and the proper functioning of neural circuits. Antibodies against LGI1 may change these physiological processes, leading to severe neurological symptoms, including cognitive decline and seizures. The primary symptoms of anti-LGI1 encephalitis include memory decline, epileptic seizures, faciobrachial dystonic seizures (FBDS), and psychiatric manifestations such as depression, sleep disturbances, and abnormal thoughts and behaviors (1–3). The clinical diagnosis of anti-LGI1 encephalitis relies on a combination of characteristic clinical manifestations, supportive diagnostic tests, and antibody detection. The definitive indicator of an autoimmune response against the LGI1 protein is the presence of LGI1 antibodies in the blood serum or cerebrospinal fluid (CSF) (2). Conventional MRI often reveals T1/T2 hyperintensities in the medial temporal lobe, hippocampus, and basal ganglia in anti-LGI1 encephalitis patients. However, MRI findings are often inconspicuous or unremarkable in a substantial proportion of affected individuals (4–7). Therefore, identifying and developing refined biomarkers that characterize structural and functional brain alterations in anti-LGI1 encephalitis is pivotal for facilitating early diagnosis and guiding effective treatment strategies for this condition.

The neuroimaging landscape of LGI1 antibody encephalitis is continually expanding, with anatomical and functional MRI (fMRI) studies revealing alterations in cortical connectivity and volume (3–5). T2/fluid-attenuated inversion recovery (FLAIR) hyperintensity in the medial temporal lobe is a common manifestation on the conventional MRI and bilateral temporal lobe hypermetabolism on positron emission tomography (PET) in patients with anti-LGI1 encephalitis (8). The resting-state fMRI scans using group independent component analysis approach demonstrate enhanced functional connectivity within the default mode network, visual network, and sensorimotor network in patients with anti-LGI1 encephalitis compared with controls (4). FDG-PET studies consistently show hypermetabolism in the primary motor cortex, suggesting its involvement in LGI1 antibody-mediated pathogenesis (6). A previous study using fMRI and diffusion tensor imaging have consistently reported alterations in both functional and effective connectivity in brain regions, including the frontal and temporal gyri, cingulate cortex, and supplementary motor area in patients. They also detected microstructural integrity impairments in widespread white matter regions, further underscoring the complex neurologic changes that occur in these individuals (9). Collectively, these functional and structural alterations in cortical regions are associated with memory, motor, and cognitive dysfunction in anti-LGI1 encephalitis.

The subcortical alterations have been observed in patients with anti-LGI1 such as the hippocampus and basal ganglia (1, 7, 10). T2/FLAIR hyperintensities and volume atrophy within the hippocampus, including its subregions such as cornu ammonis 2/3 (CA2/3) and CA4/dentate gyrus, have been consistently observed in patients with anti-LGI1 encephalitis (5, 11–14). Furthermore, T1 hyperintensity in the basal ganglia on MRI scans and hypermetabolism detected through FDG-PET have emerged as clinically valuable biomarkers, particularly in cases associated with FBDS (7, 8, 15). In contrast, FreeSurfer and voxel-based morphometry analyses have failed to demonstrate substantial differences in basal ganglia and cortical gray matter volumes (5). Notably, a majority of existing studies primarily rely on volumetric analyses, limiting their ability to detect subtle alterations within brain regions. To gain precise understanding of subtle subcortical structural alterations, we employed shape analysis which is capable of capturing localized shape deformations in anti-LGI1 encephalitis using high-resolution anatomical MRI data. We hypothesized that the shape deformations of subcortical structures might be related to clinical variables in patients with encephalitis.

## 2 Materials and methods

### 2.1 Participants

This retrospective study included 31 anti-LGI1 encephalitis patients and 31 normal controls. Clinical and imaging data were extracted from medical records of patients who had already undergone MRI scans prior to immunotherapy initiation. All data were anonymized and analyzed *post hoc* after approval by the Qilu Hospital of Shandong University Ethics Committee. All participants provided written informed consent for MRI scans and data usage. The diagnostic criteria were based on clinical symptoms and the detection of specific anti-LGI1 antibodies in serum and cerebral spinal fluid, as reported in a previous study (16). The clinical symptoms included memory and cognitive impairment, seizures, FBDS, and behavioral abnormalities. The severity of symptoms was assessed for all patients using the modified Rankin Scale (mRS). Furthermore, mental and cognitive abilities of the patients were evaluated using the Mini-Mental State Examination (MMSE), the Montreal Cognitive Assessment (MoCA), and word immediate and delayed recall tests. Detailed clinical characteristics is demonstrated in **Table 1**.

**Table 1:** Clinical characteristics of patients with anti-LGI1 encephalitis.

Characteristic	No.
Age, mean (SD)	54.87 (13.56) years
Sex	22 men and 9 women
Modified Rankin Scale score ^a^, mean (SD)	2.10 (0.75)
Time from symptom onset to diagnosis, mean (SD)	3.5 (2.2) months
Mini-Mental State Examination (MMSE), mean (SD)	21.27 (6.68)
Montreal Cognitive Assessment (MoCA), mean (SD)	18.07 (7.40)
Immediate recall in MMSE	2.34 (0.86)
Delayed recall in MMSE	1.66 (1.08)
Delayed recall in MoCA	1.55 (1.66)
Symptom	
Memory impairment	24/31 (77%)
Seizure	23/31 (74%)
Faciobrachial dystonic seizures	13/31 (42%)
Cerebrospinal fluid	
Protein ^b^ (g/L), mean (SD)	0.44 (0.30)
Lactic Acid ^c^ (mmol/L), mean (SD)	2.05 (0.37)
Antibodies to LGI1	
Serum (positive)	31/31 (100%)
Cerebrospinal fluid (positive)	22/26 (85%)

LGI1, Leucine-rich glioma-inactivated 1; SD, standard deviation.

### 2.2 Image acquisition

Structural MRI (sMRI) was performed on a Siemens Verio 3.0 Tesla MRI scanner (Siemens, Erlangen, Germany) using a 32-channel head coil at the Qilu Hospital of Shandong University. The head motion was minimized by securing the heads of the participants in foam pads in the supine position. T1-weighted high-resolution anatomical images were acquired for each participant using a 3-dimensional magnetization-prepared rapid acquisition gradient echo pulse sequence. The imaging parameters were as follows: repetition time (TR) = 2000 milliseconds; echo time (TE) = 2.34 milliseconds; inversion time = 900 milliseconds; matrix size = 256 × 256; number of slices = 192; voxel size = 1 × 1 × 1 mm^3^; flip angle = 9°; and slice thickness = 1.0 mm.

### 2.3 Subcortical shape analysis

The sMRI images were preprocessed using the Manual Computational Anatomy (CAT12) toolbox, which included skull stripping and bias correction (17). The intensity nonuniformity was measured and corrected using an automatic bias field correction method based on the Gaussian mixture model. We employed the FMRIB Software Library (FSL) FIRST (18) segmentation tool, which is based on a Bayesian model, to segment the skull-stripped anatomical images. A total of 15 subcortical structures, including the bilateral hippocampus, nucleus accumbens (NAc), putamen, caudate, pallidum, thalamus, amygdala, and brainstem, were obtained using this tool. Quality control was ensured by visually inspecting these 15 subcortical segmentations. The mesh representations of the segmentations were computed through linear combinations of shape modes of variation. Specifically, the scalar projection values were estimated by projecting the vertex locations from each participant onto the surface normal of the average shape, where a positive value represented an outward deviation from the surface and a negative value represented an inward deviation. All meshes were aligned to the mean shape from the shape model.

### 2.4 Volumetric analysis

The volumetric representations of the fifteen subcortical segmented structures were created by identifying the mesh passed voxels and filling the estimated surface meshes as well as boundary correction which ensured that there was no overlap between structures. The label numbers of each subcortical structure were determined by the standard labels of the color table built in FSLView. The volume and the number of voxels were measured with the threshold determined by the desired label number. The two sample t-test was utilized to compare subcortical volumes with age and sex as covariates to investigate the group differences between patients and controls. False discovery rate (FDR) correction for multiple comparisons was used to assess the significant volumetric alterations.

### 2.5 Statistical analysis and correlations with clinical scores

The vertex-wise statistics was performed for investigating localized shape differences. The randomize in FSL toolbox was utilized for conducting the group statistical analysis between patients at the acute stage and normal controls, with age, gender, and disease duration as covariates. The multiple comparison correction was performed using the threshold-free cluster enhancement (TFCE) with family-wise error (FWE) correction (*P* <.05). The inference was carried out by running 5000 random permutations when building the null distribution. Furthermore, Pearson correlation was computed to assess the relationship between clinical disease severity (mRS), cognitive scores (MMSE and MoCA), and shape measures of the 15 subcortical structures based on the mean value of the significant regions obtained from the shape analyses.

## 3 Results

### 3.1 The demographic characteristics of the patient and control groups

There were 31 patients with anti-LGI1 encephalitis (22 males and 9 females; mean age 54.9 ± 13.6 years) and 31 normal controls (20 males and 11 females; mean age 52.9 ± 11.3 years). There were no statistically significant differences in age or gender between the patient and control groups.

### 3.2 Subcortical shape abnormalities between the patient and control groups

**Figure 1:**
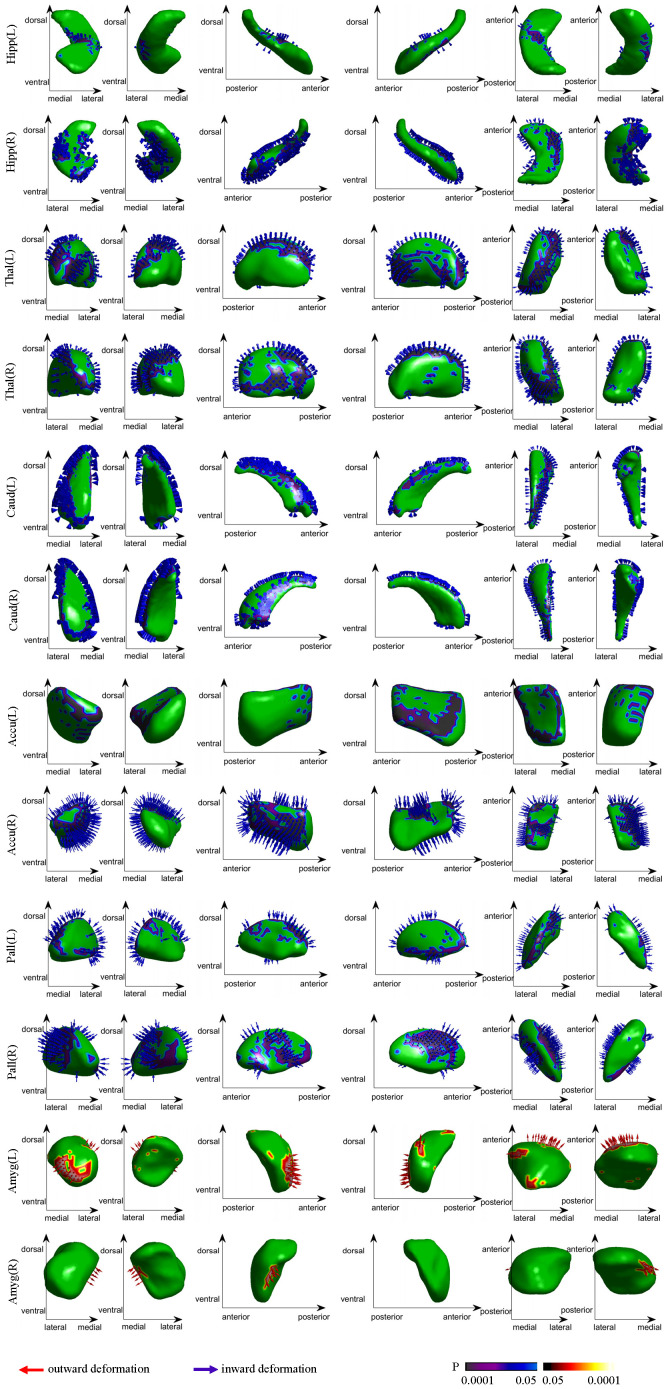
Group differences in the subcortical structural shape analyses (patients vs controls). The blue arrows indicate notable inward shape deformations, and the red arrows indicate the notable outward shape deformations in patients compared with controls.

Notable inward shape deformations were detected in patients with anti-LGI1 encephalitis compared with normal controls in the bilateral hippocampus, NAc, caudate, and thalamus. In contrast, remarkable outward shape deformations were noted in the bilateral amygdala with TFCE-FWE correction at *P* <.05, as illustrated in **Figure 1**. The detailed locations and voxel clusters are listed in **Table 2**. The mean and standard deviation of the shape measurements for each subcortical structure were also analyzed for global quantification, as shown in **Table 3**. The group comparison of brain volumetric differences between patients and controls were shown in **Table 4**. The volumes in the right hippocampus and right nucleus accumbens in anti-LGI1 encephalitis group were significantly decreased than that of the control group.

**Table 2:** Comparisons of shape deformations of subcortical brain regions between patients with anti-LGI1 encephalitis and normal controls.

Brain regions	Side	Peak location			T statistic	*P* value	Number of vertices
		*x*	*y*	*z*			
Patients vs Controls (negative)							
Thalamus	L	−13	−4	10	3.94	.0044	1939
	R	1	−16	4	4.46	.0004	2641
Hippocampus	L	−22	−29	−9	4.35	.003	276
	R	32	−25	−8	5.57	.0002	1880
Caudate	L	−17	−18	24	3.47	.005	974
	R	12	−3	20	3.71	.002	1769
Pallidum	L	−21	1	−1	3.27	.008	675
	R	19	−10	−4	3.76	.0126	372
Nucleus accumbens	L	−12	9	−8	4.42	.0002	572
	R	5	10	−4	4.53	.0002	460
Patients vs Controls (positive)							
Amygdala	L	−14	−9	−20	3.82	.016	91
	R	19	−5	−24	4.18	.015	28

LGI1, Leucine-rich glioma-inactivated 1.

**Table 3:** The mean shape measures of the subcortical brain regions between patients with LGI1 encephalitis and normal controls.

Brain regions	Patients (mean±std)	Controls (mean±std)	T value^*^	P value ^**^
Left Hippocampus	-0.088±0.203	0.079±0.099	-3.29	**0.0049**
Right Hippocampus	-0.113±0.171	0.101±0.072	-5.11	**0.0001**
Left Nucleus accumbens	-0.180±0.352	0.162±0.151	-3.96	**0.0017**
Right Nucleus accumbens	-0.198±0.470	0.178±0.178	-3.33	**0.0049**
Left Caudate	-0.106±0.281	0.095±0.144	-2.82	**0.0102**
Right Caudate	-0.089±0.204	0.080±0.111	-3.21	**0.0053**
Left Putamen	-0.105±0.322	0.095±0.166	-2.43	**0.0231**
Right Putamen	-0.081±0.170	0.073±0.149	-2.98	**0.0086**
Left Pallidum	-0.064±0.189	0.058±0.229	-1.78	0.0831
Right Pallidum	-0.049±0.109	0.044±0.186	-1.85	0.0775
Left Thalamus	-0.067±0.128	0.061±0.144	-2.88	**0.0100**
Right Thalamus	-0.068±0.115	0.061±0.123	-3.33	**0.0049**
Left Amygdala	0.226±0.378	-0.203±0.375	3.51	**0.0046**
Right Amygdala	0.200±0.286	-0.180±0.158	5.14	**0.0001**
Brainstem	0.086±0.191	-0.077±0.168	2.80	**0.0102**

^*^Negative T value presents smaller mean shape measure of the brain region in patients.

^**^False discovery rate (FDR) correction for multiple comparisons.

Bold values denote significant differences (p < 0.05).

**Table 4:** Volumetric changes of the subcortical brain regions.

Brain regions	Patients (mean±std) mm^3^	Controls (mean±std) mm^3^	T value^*^	P value^**^
Left Hippocampus	4826.8±564.3	5152.8±260.4	-2.33	0.0645
Right Hippocampus	5182.5±485.2	5683±282.6	**-3.93**	**0.0055**
Left Nucleus accumbens	730.9±133.6	821.9±64.5	-2.72	0.0501
Right Nucleus accumbens	571.4±135.9	675.9±71.5	**-3.01**	**0.0359**
Left Caudate	4598.8±352.2	4819.6±213.6	-2.36	0.0645
Right Caudate	5030.9±308.6	5249.1±239.3	-2.45	0.0645
Left Putamen	6961.4±593.3	7034.5±286.1	-0.49	0.6711
Right Putamen	6868.8±325.1	6874.3±359.4	-0.05	0.9608
Left Pallidum	2363.3±162.7	2450.2±265.4	-1.19	0.2977
Right Pallidum	2384.1±117.4	2460.7±227.2	-1.28	0.2830
Left Thalamus	10499.3±189.4	10584.8±183.1	-1.42	0.2741
Right Thalamus	10087.2±194.1	10217.4±206.1	-1.99	0.1142
Left Amygdala	2362.9±234.8	2247.1±230.9	1.53	0.2518
Right Amygdala	2106.5±274.2	2066.6±121.9	0.59	0.6450
Brainstem	30486.3±745.9	30184.9±663.8	1.32	0.2830

^*^ Negative T value means smaller volumes of the brain region in patients.

^**^ False discovery rate (FDR) correction for multiple comparisons.

Bold values denote significant differences (p < 0.05).

### 3.3 Correlations between shape and clinical measures

**Figure 2:**
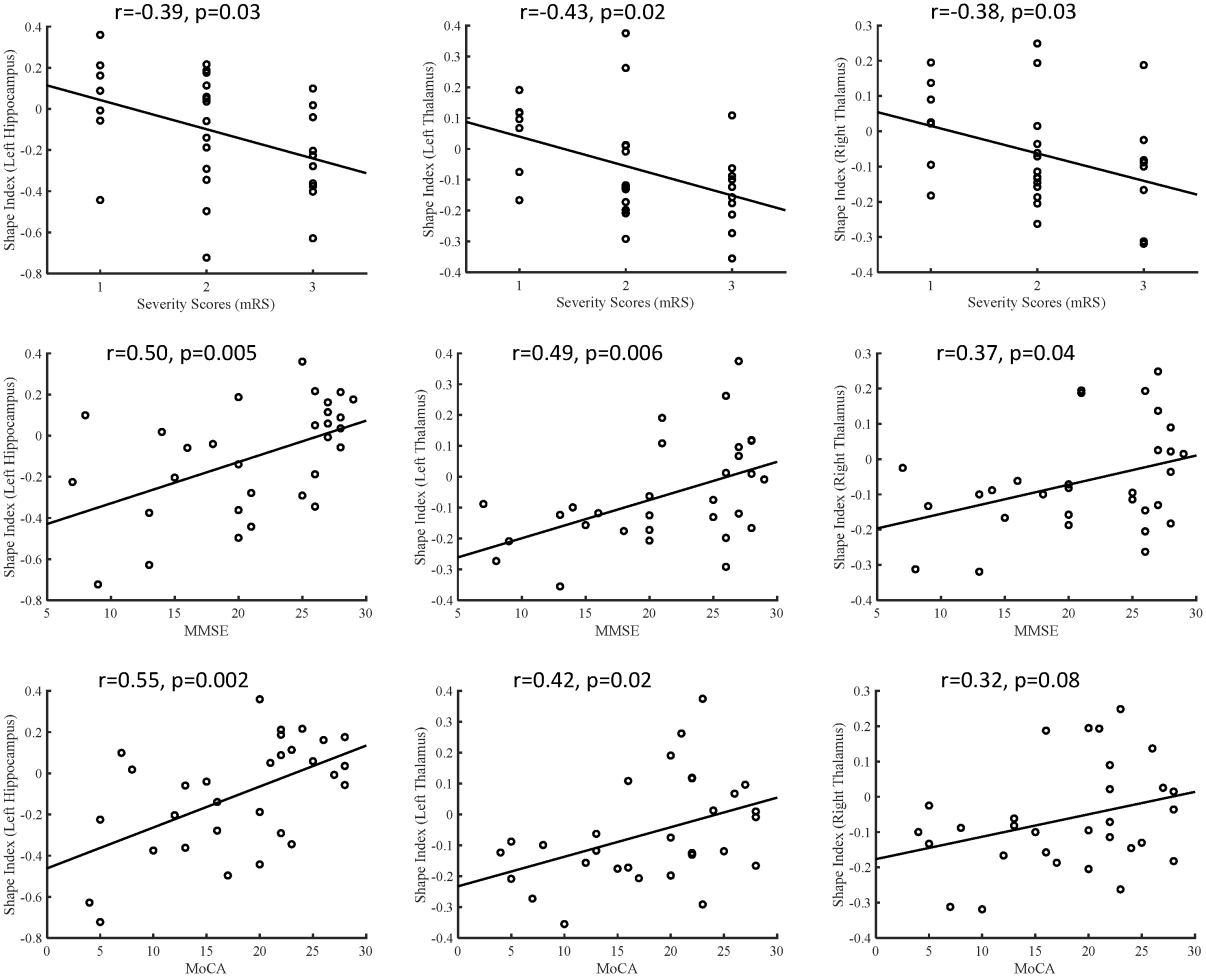
Correlations between the subcortical shape measurements and clinical disease severity and cognitive function, including shape measurements versus mRS, shape measurements versus MMSE, and shape measurements versus MoCA. MMSE, Mini-Mental State Examination; MoCA, Montreal Cognitive Assessment.

In the anti-LGI1 encephalitis group, the subcortical shape measurements in the thalamus and hippocampus exhibited inverse correlations with the mRS scores but positive correlations with cognitive scores (MMSE and MoCA), as depicted in **Figure 2**. Greater inward deformations in the hippocampus and thalamus correlated with higher mRS scores and lower MMSE/MoCA scores, indicating associations with disease severity and cognitive impairment. In addition, the hippocampus and thalamus exhibited significant positive correlations with cognitive scores, including immediate recall in MMSE (hippocampus: *r* = 0.37, *P* = 0.049; thalamus: *r* = 0.45, *P* = 0.015), delayed recall in MMSE (hippocampus: *r* = 0.54, *P* = 0.002; thalamus: *r* = 0.40, *P* = 0.03), and delayed recall in MoCA (hippocampus: *r* = 0.48, *P* = 0.009; thalamus: *r* = 0.38, *P* = 0.04). Furthermore, we examined the correlations between subcortical volumes and clinical assessments. Our analysis revealed that reduced volume in the left thalamus was significantly associated with poorer clinical outcomes, showing negative correlation with mRS scores (*r* = -0.48, *P* = 0.02) and positive correlations with both MMSE (*r* = 0.45, *P* = 0.03) and MoCA scores (*r* = 0.44, *P* = 0.03). No other subcortical structures demonstrated significant volume-clinical correlations. These findings suggest that shape analysis may offer greater sensitivity than volumetric measurements in detecting clinically relevant morphological changes.

## 4 Discussion

This study investigated subcortical shape abnormalities in patients with anti-LGI1 encephalitis using advanced shape analysis. Our results highlighted inward shape deformations in bilateral subcortical structures, including the hippocampus, nucleus accumbens, caudate, pallidum, and thalamus in patients group. Additionally, correlation analyses revealed a significant association between greater inward shape indices in the hippocampus and thalamus and increased disease severity and cognitive impairment. Therefore, precise localization of these subcortical structural alterations in anti-LGI1 encephalitis holds significant potential as vital biomarkers for elucidating the clinical presentations and neuropsychological consequences of this condition.

A growing body of evidence has pinpointed the precise structural locations within the hippocampus and thalamus, the key components of the Papez circuit, in relation to various neurologic conditions (19–22). The Papez circuit is a fundamental pathway of the limbic system, encompassing a series of interconnected structures such as the hippocampus, thalamus, cingulate cortex, mammillary body, mammillothalamic fibers, and fornix. These structures are vital for spatial learning, episodic memory, and emotional processing (20, 23) Notably, the hippocampus serves as both the starting and end points of this circuit, playing a pivotal role in memory formation. In the context of anti-LGI1 encephalitis, the hyperexcitability of the hippocampus due to autoimmune effects on LGI1 has emerged as a primary target of this disease (6). Our study contributes to this understanding by presenting inward shape deformations of the hippocampus in patients with anti-LGI1 encephalitis compared with healthy controls. This finding aligns with previous research demonstrating hippocampal T2/FLAIR hyperintensities, reduced volumes on MRI, increased metabolism on PET, and impaired microstructural integrity in other forms of encephalitis, such as gamma aminobutyric acid B (GABA-B) antibody encephalitis (24, 25). Notably, our analysis revealed localized inward deformations on the surface of the intermediate and ventral hippocampus, particularly in the lateral and medial regions, which have not been previously reported in volumetric studies. Additionally, we observed inward shape deformation in the bilateral thalamus of patients, a finding that has not been previously documented in volumetric analyses (5, 11). These focal shape alterations may be more sensitive indicators of disease than overall regional volume changes, suggesting that subtle changes in subfields can have considerable implications (26, 27). The anterior thalamus is a critical relay node in the Papez circuit, which is responsible for receiving and processing information from mammillothalamic fibers to the cingulum (20, 21). The observed shape deformation of the anterior thalamus in this study underscores its influence on episodic memory, a function that has been implicated in other neurologic disorders (21, 28–30). Furthermore, we observed an obvious negative correlation between the shape index in the thalamus and disease severity, indicating that an increase in shape deformations may be associated with more severe disability among patients. Collectively, these deformations in the thalamus and hippocampus indicate disruptions within the Papez circuit, which is known to be vulnerable to encephalitis and can lead to memory impairment. The localized involvement of the thalamus in our findings offers valuable insights into the diagnosis and treatment of anti-LGI1 encephalitis. Thus, the specific structural alterations within the Papez circuit need to be considered when evaluating patients with this disease, and it may help develop more targeted therapeutic approaches. Moreover, Krohn et al. (31) identified widespread disruptions in white matter connectivity networks in anti-LGI1 encephalitis patients, particularly affecting the hippocampus, caudate, nucleus accumbens, thalamus, and multiple neocortical regions. Our observation of localized shape deformations in the hippocampus and thalamus not only corroborates these findings but further extends them by demonstrating specific patterns of subcortical structural alteration at higher spatial resolution. This convergence of evidence from both connectivity and morphometric analyses strongly implicates these limbic structures as key targets of LGI1 autoimmunity.

Additionally, our correlation analysis demonstrated a notable link between elevated inward shape indices in the hippocampus and thalamus, and the exacerbation of disease severity alongside impaired cognitive function. This finding underscores the need for monitoring these subcortical regions in patients with anti-LGI1 encephalitis, as they may serve as valuable biomarkers for assessing disease progression and cognitive outcomes. This finding aligns with a previous study that indicated a decrease in the hippocampal volume, coupled with higher mRS scores, among patients diagnosed with anti-LGI1 encephalitis (5). Clinicians can track changes in the shape indices of these regions over time to identify patients who are at risk for more severe disease manifestations and develop targeted interventions to prevent or mitigate these consequences.

Furthermore, we identified inward deformations on the surface of the caudate, a crucial component of the basal ganglia and motor circuit, in patients with anti-LGI1 encephalitis compared with normal controls. These deformations were not readily apparent on routine MRI scans but were evident in FDG-PET scans, which revealed hypermetabolism of the basal ganglia (4, 5, 15). The basal ganglia play a pivotal role in motor control, influencing both the facilitation and inhibition of behavior, as well as movement regulation. As one of the primary input nuclei for the basal ganglia, the caudate receives information from the cortex through the corticostriatal pathway, contributing effectively to motor function (32). Our findings of shape deformations in the caudate support its vital role in disorders related to the initiation or suppression of movements, which may explain the motor seizures and FBDS observed in anti-LGI1 encephalitis. Therefore, structural alterations in the basal ganglia, particularly the caudate, should be considered when diagnosing and treating this disease.

The NAc serves as a pivotal limbic-motor interface and a critical node in seizure transmission. We observed inward shape deformations in the shell of the NAc in patients with anti-LGI1 encephalitis compared with healthy controls. Previous studies have emphasized the crucial role of NAc, particularly its shell region, in seizure propagation and epileptogenesis, particularly in the context of mesial temporal lobe epilepsy (mTLE) (33). Specifically, the mu opioid receptors in the NAc have been implicated in the postictal reduction of locomotion following amygdaloid-induced seizures in rats (34). Moreover, studies have demonstrated abnormal structural and functional connectivity within the core and shell subdivisions of the NAc in patients with mTLE (33, 35). Notably, clinical studies have demonstrated that electrical deep brain stimulation of the NAc can substantially reduce seizure severity (36). In line with these findings, our results suggested that anatomical damage to the NAc shell might disrupt the intricate link between limbic and motor systems, contributing to epileptogenesis and seizure propagation in anti-LGI1 encephalitis. Thus, it is vital to consider the role of the NAc in the pathogenesis and treatment of this disease.

This study identified notable outward shape deformations in the bilateral amygdala of patients with anti-LGI1 encephalitis. This phenomenon has been previously observed in mTLE and associated with amygdala enlargement, which is considered a potential seizure focus (37, 38). Animal studies have reported the kindling phenomenon in the amygdala, which can induce seizures and contribute to epileptogenesis (34, 39). Clinical investigations have further demonstrated that amygdalotomy, or surgical transection of the amygdala, can effectively control seizures and lead to freedom from seizure in patients with temporal lobe epilepsy (40), including those with antibody-associated limbic encephalitis (41). Additionally, a recent study revealed that a structural network topology in limbic encephalitis was associated with amygdala enlargement (42). During the acute stage, the permeability of vessels increased rapidly and causes edema of the tissues which might lead to the amygdala enlargement. Therefore, our surface deformation results, in conjunction with previous findings, reinforce the crucial role of the amygdala in seizure occurrence and transmission in anti-LGI1 encephalitis. These insights contribute to a more comprehensive understanding of the disease and guide the development of targeted therapies.

The observed inward deformations in the hippocampus and thalamus not only correlate with clinical severity but also align with the known pathophysiology of anti-LGI1 encephalitis, where LGI1 antibody-mediated disruption of synaptic transmission preferentially targets these regions. Given that these changes are detectable even in the absence of volumetric loss, shape analysis could serve as an early biomarker for subclinical disease progression, particularly in seropositive patients with nonspecific MRI findings. Future studies will investigate whether these shape biomarkers can predict treatment responsiveness. For instance, baseline deformation severity might identify patients requiring aggressive immunotherapy, while serial scans could monitor therapeutic efficacy. This approach could complement existing tools like CSF antibody titers, which lack spatial specificity.

This study had several limitations. First, the relatively small sample size limited the generalizability of the findings, and hence further studies with larger cohorts are necessary to validate and strengthen the conclusions. Second, the complexity of the disease necessitates a comprehensive understanding of its manifestations across various imaging modalities, including structural, functional, and metabolic changes. Therefore, adopting multimodal imaging approaches is crucial for identifying more reliable and effective biomarkers to improve disease diagnosis and management. Future work will integrate multimodal neuroimaging to unravel structure-function relationships. For example, combining shape analysis with resting-state fMRI could clarify whether hippocampal deformations disrupt functional connectivity in memory networks. The PET-MRI fusion may reveal whether localized deformations correlate with regional hypermetabolism, particularly in the basal ganglia or medial temporal lobe. Diffusion tensor imaging may assess if shape changes coincide with white matter tract damage, linking structural pathology to cognitive deficits. These multimodal approaches may yield biomarkers for early diagnosis, subtype classification, and treatment monitoring. Third, although our cohort was unified by treatment naïve status, post-immunotherapy follow-up data are needed to assess whether shape metrics normalize with clinical improvement. This would clarify if deformations are reversible or reflect permanent damage. Finally, subtype specific investigations stratifying patients by predominant symptoms could reveal distinct neuroanatomical signatures. Larger cohorts will enable machine learning approaches to classify subtypes based on shape biomarkers. Symptom severity stratification may uncover differential biomarker utility, guiding personalized therapeutic strategies.

In conclusion, this study demonstrates that the shapes of subcortical structures serve as reliable biomarkers indicating damage to the limbic system and basal ganglia in patients with anti-LGI1 encephalitis. These subcortical shape abnormalities are significantly linked to symptom severity and cognitive function deficits, underscoring their clinical relevance. The association between the extent of inward shape alterations in the hippocampus and thalamus and the degree of disease severity and cognitive impairment highlights the potential of these biomarkers in assessing disease progression and therapeutic response. Therefore, subcortical shape analysis not only enhances our understanding of the neuroanatomical damage associated with anti-LGI1 encephalitis but also offers valuable tools for improving diagnosis, monitoring, and treatment strategies.

## Data Availability

The original contributions presented in the study are included in the article/**Supplementary Material**. Further inquiries can be directed to the corresponding authors.
